# Carpal compression, Phalen’s and Tinel’s test: Which one is more suitable for carpal tunnel syndrome?

**Published:** 2016-07-06

**Authors:** Mostafa Almasi-Doghaee, Reza Boostani, Morteza Saeedi, Saeed Ebrahimzadeh, Amir Moghadam-Ahmadi, Mohammad Javad Saeedi-Borujeni

**Affiliations:** 1 Department of Neurology, School of Medicine, Iran University of Medical Sciences, Tehran, Iran; 2 Department of Neurology, School of Medicine, Mashhad University of Medical Sciences, Mashhad, Iran; 3 Department of Biostatistics, School of Health, Mashhad University of Medical Sciences, Mashhad, Iran; 4 Department of Neurology, School of Medicine, Rafsanjan University of Medical Sciences, Rafsanjan, Iran; 5 Department of Anatomical Sciences, School of Medicine, Neurosciences Research Center, Alzahra Hospital, Isfahan University of Medical Sciences, Isfahan, Iran

**Keywords:** Carpal Tunnel Syndrome, Phalen’s Test, Tinel’s Test, Carpal Compression Test

One of the main causes of hand dysfunction is carpal tunnel syndrome (CTS) and because of its high occurrence, early diagnosis is very important and may reduce disability caused by this condition.^1^ In addition to paraclinical procedures including electrodiagnostic (EDX) studies and median nerve sonography,^2^ a variety of clinical tests have been suggested for assessment of CTS among them, Tinel’s test (TT) and Phalen’s test (PT) are the most popular ones.^3^

Previous studies revealed differences in sensitivity and specificity with values of 61-91 percent and 33-93 percent for the PT, and 41-74 percent and 80-91 percent for the TT, respectively.^3^ Carpal compression test (CCT) was introduced recently and had greater sensitivity and specificity than TT and PT in some studies.^4^^,^^5^ In this study, we aimed to compare the efficacy of CCT with TT and PT in diagnosis of CTS.

In our study, all the patients suspected to suffer from CTS referred to the electrodiagnostic ward of Ghaem Hospital (Mashhad, Iran) from 2011 to 2012 were included. After taking detailed medical history and performing physical examination including the PT, TT and CCT, all the data were gathered in a checklist for each patient. EDX studies were conducted and definitive diagnosis of CTS was based on the results (by EDX criteria). Patients were divided into two groups of CTS and non-CTS. 

Statistical analysis was performed using independent-sample t and chi-square tests via SPSS software (version 15, SPSS Inc., Chicago, IL, USA). P-value of less than 0.05 was accepted as statistically significant. The sensitivities and specificities of the PT, TT and CCT were gained via comparison with the electrodiagnostic tests, as gold standard. 

Of 89 patients, CTS was diagnosed in 80.9%. In terms of gender, age and employment, there was no statistical significant difference between the CTS and non-CTS groups (P > 0.050 for all). 

The frequency of CCT-positive patients was statistically greater in CTS group (80.6%) than non-CTS one (47.1%) (P = 0.008). The frequency of the PT-positive patients was greater in non-CTS group (59.7 vs. 64.7%) with no statistically significant difference (P = 0.464). 

The calculated sensitivity and specificity were 80.6 and 52.9% for CCT, 59.7 and 35.3% for PT, and 65.3 and 47.1% for TT, respectively. 

In receiver operating characteristics (ROC) curve analysis, the area under the curve (AUC) was 0.67 (95% CI: 0.51-0.82, P = 0.032) for CCT, 0.56 for TT and 0.48 for PT (P > 0.050 for both) (Figure 1). 

**Figure 1:**
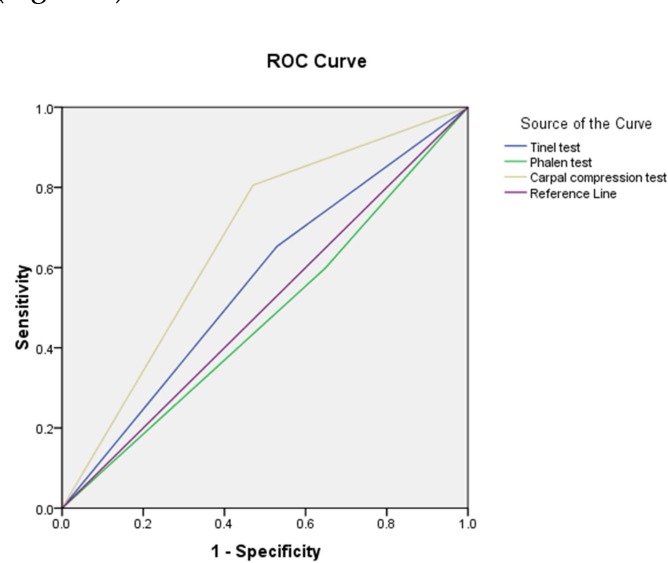
Receiver operating characteristics (ROC) curve analysis of carpal compression test (CCT), Tinel’s test (TT) and Phalen’s test (PT) for diagnosis of carpal tunnel syndrome (CTS)

In non-CTS group, there was a moderate negative agreement between the TT and CCT (k = -0.524; P = 0.030). There was a moderate positive agreement between the PT and TT in CTS group (k = 0.409; P < 0.001).

In our study, TT and CCT were more positive in CTS group and PT was more positive in non-CTS group but just positive CCT was statistically different between CTS and non-CTS groups. ROC cure analysis showed the accuracy of CCT for diagnosis of CTS is higher than TT and PT. AUC for TT and PT were around 0.5 which means that these tests cannot help us in depicting patients with CTS. Previous studies reported contradictory results about the sensitivity and specificity of CCT, but in most of them, they were greater than the sensitivities and specificities of the TT and PT.^4^^,^^5^ Our results confirm the importance of CCT in diagnosis of CTS.

One another point is that the moderate negative agreement between the TT and CCT in non-CTS group means that both the tests would rarely be positive among them. In other words, if both CCT and TT were positive, the diagnosis is more likely to be CTS. In addition, the positive agreement between the PT and TT in CTS group means that performing one of these tests is sufficient for patients suspected to suffer from CTS; because if one of them is positive, it is very probable that the other one is also positive and vice versa.

In conclusion, as the sensitivity and specificity of the CCT are greater than those of the TT and PT, we recommend routine use of CCT for screening the patients suspected to CTS.

## Notes:

**How to cite this article:** Almasi-Doghaee M, Boostani R, Saeedi M, Ebrahimzadeh S, Moghadam-Ahmadi A, Saeedi-Borujeni MJ. Carpal compression, Phalen’s and Tinel’s test: Which one is more suitable for carpal tunnel syndrome? Iran J Neurol 2016; 15(3): 173-4.
